# A Personal Statement

**Published:** 1914-01

**Authors:** 


### A Personal Statement.

The editor has tried to avoid, so far as possible, anything approaching self-advertisement in the JOURNAL, but he may be permitted to dispel certain negative impressions. Today a medical friend telephoned, before referring a patient, to ask if the editor was still in practice. As something of the same sort has been alluded to before, more or less jestingly, it may be stated that the proceeds of medical journalism do not in this or most other cases warrant retirement from practice and that, on the whole, active medical interests are a wholesome restraint on the tendency toward the attitude of a man at the desk. In fact, in the early months of the present management of the JOURNAL it was quite necessary that the editor should have a practice, for the sake of the JOURNAL itself. It is scarcely conceivable that any medical journal of comparable field can be made a source of income to any great extent and, if it could be, the inclination of the present incumbent would be to continue in active practice until such time as one may be allowed to retire without loss of the self-respect which comes from work.

